# Cold Atmospheric Plasma induces accumulation of lysosomes and caspase-independent cell death in U373MG glioblastoma multiforme cells

**DOI:** 10.1038/s41598-019-49013-3

**Published:** 2019-09-09

**Authors:** Gillian E. Conway, Zhonglei He, Ana Lacramioara Hutanu, George Paul Cribaro, Eline Manaloto, Alan Casey, Damien Traynor, Vladimir Milosavljevic, Orla Howe, Carlos Barcia, James T. Murray, Patrick J. Cullen, James F. Curtin

**Affiliations:** 1 School of Food Science & Environmental Health, Technological University Dublin, Dublin, Ireland; 2 FOCAS Research Institute, Technological University Dublin, Dublin, Ireland; 3 Environmental Sustainability & Health Institute, Technological University Dublin, Dublin, Ireland; 4 School of Physics & Clinical & Optometric Sciences, Technological University Dublin, Dublin, Ireland; 5 School of Biological & Health Sciences, Technological University Dublin, Dublin, Ireland; 6 In-Vitro Toxicology Group, Institute of Life Science, Swansea University Medical School, Swansea University, Singleton Park, Swansea, UK; 7 School of Biochemistry & Immunology, Trinity Biomedical Sciences Institute, Trinity College Dublin, Dublin, Ireland; 8 Institut de Neurociències & Department of Biochemistry and Molecular Biology, School of Medicine, Universitat Autònoma de Barcelona, Barcelona, Spain; 9 School of Chemical and Biomolecular Engineering, University of Sydney, Darlington, Australia

**Keywords:** CNS cancer, Apoptosis

## Abstract

Room temperature Cold Atmospheric Plasma (CAP) has shown promising efficacy for the treatment of cancer but the exact mechanisms of action remain unclear. Both apoptosis and necrosis have been implicated as the mode of cell death in various cancer cells. We have previously demonstrated a caspase-independent mechanism of cell death in p53-mutated glioblastoma multiforme (GBM) cells exposed to plasma. The purpose of this study was to elucidate the molecular mechanisms involved in caspase-independent cell death induced by plasma treatment. We demonstrate that plasma induces rapid cell death in GBM cells, independent of caspases. Accumulation of vesicles was observed in plasma treated cells that stained positive with acridine orange. Western immunoblotting confirmed that autophagy is not activated following plasma treatment. Acridine orange intensity correlates closely with the lysosomal marker Lyso TrackerTM Deep Red. Further investigation using isosurface visualisation of confocal imaging confirmed that lysosomal accumulation occurs in plasma treated cells. The accumulation of lysosomes was associated with concomitant cell death following plasma treatment. In conclusion, we observed rapid accumulation of acidic vesicles and cell death following CAP treatment in GBM cells. We found no evidence that either apoptosis or autophagy, however, determined that a rapid accumulation of late stage endosomes/lysosomes precedes membrane permeabilisation, mitochondrial membrane depolarisation and caspase independent cell death.

## Introduction

Glioblastoma multiforme (GBM), a grade IV malignant astrocytoma, is highly proliferative, invasive and resistant to most therapies. GBM is generally treated using a combination of physical, chemical and biological treatments. Surgical interventions and radiation therapy in combination with either pharmaceuticals or biopharmaceuticals have been the mainstay of cancer treatment for decades^1–3^. While marginal gains are evident in 1 and 2-year survival rates, five-year prognosis for patients diagnosed with GBM remains almost completely refractory to treatment.

In recent years, advances in materials and technologies have led to the possibility of new physical interventions for the treatment of cancer either alone (drug-free) or in combination with existing therapeutic modalities^4,5^. One promising advance is the development of Cold Atmospheric Plasma (also known as cold plasma, atmospheric pressure plasma, atmospheric cold plasma and non-thermal atmospheric plasma) that can operate at or below the temperature of human tissue, opening a wide range of biological and biomedical applications without risking thermal damage. Of particular interest is the possible use of Cold Atmospheric Plasma, referred to simply as plasma herein, to induce cell death preferentially in tumour cells. Plasma is the fourth state of matter and directly interacts with biological material, cells and tissues through chemical and physical effects. This emerging field of study, called plasma medicine, aims to use physical plasmas for therapeutic applications and has been subject to intensive study over the last decade.

A major advantage of plasma over conventional therapies as an anti-tumour agent is the broad range of biological responses that are initiated, reducing the likelihood for resistance to develop. The generation of short- and long-lived reactive species, generation of photons, heat, pressure gradients, charged particles, and electrostatic and electromagnetic fields have all been shown to induce biological effects^6–8^. Plasma is a partially ionized gas that contains various concentrations of free electrical charges, atoms, ions and electrons which are generated by an energy supply to a neutral gas^9^, which generates a unique physical and chemical environment when exposed to biological tissues including activating short- and long-lived reactive oxygen species (ROS)^6–8,10^ and many of which are known to induce biological effects. Many studies have shown that plasma can induce a cytotoxic response in *vitro* in a variety of cell lines, for example glioblastoma, cervical, breast, colorectal, and lung^11–15^, of which the cell death mechanisms have been reported as apoptosis^16,17^, cell cycle arrest^18,19^, autophagy^20^ and necrosis^20^ depending on the tumour model studied and the plasma device/system used. This is not unexpected given the wide array of chemical and physical alterations that plasma can induce in cells and the interconnectivity of initiation and signal transduction between different subtypes of cell death. Many studies to date have demonstrated an important role for reactive oxygen species generated by plasma treatment, including, H_2_O_2_, that induce apoptosis in glioblastoma cells as well as many other cancer cells^12,18,21,22^.

There are two main mechanisms of cell death, requiring either active processes (i.e. energy-dependent) such as apoptosis, autophagy and necroptosis or those that occur passively such as necrosis^23^. The most common and extensively studied mechanism is apoptosis, a term first used in 1972 to describe a form of cell death with distinct morphological features, which had been described more than a century previously by Rudolph Virchow^24^. Apoptosis is generally characterized by distinct morphological characteristics, however subsequent recognition that biochemical changes, such as DNA fragmentation and caspase activation underpin apoptosis and have led to a large body of literature describing apoptotic events^24^. It is widely accepted that caspases play a central role in both the intrinsic and extrinsic apoptotic pathway, but it is also noted that caspase-independent apoptosis (CICD) has also been demonstrated, and can manifest with morphological signs of apoptosis, autophagy or necrosis^25–27^. Autophagy is a highly regulated process that all eukaryotic cells carry out by sequestering damaged or defective organelles within a double-membrane bound vesicle called an autophagosome, which then fuses with a lysosome to form an autolysosome where sequestered cargo is degraded and recycled^28,29^. Autophagy is associated with both cell survival and cell death phenotypes. During physiological stress, such as nutrient deprivation, autophagy is activated to degrade organelles and proteins to provide material for essential biosynthetic pathways and energy production, therefore, sustaining cellular integrity and homeostasis^28,30^. As such, autophagy is primarily a survival signal that is first activated in cells to prevent cellular demise. However, under prolonged or overwhelming physiological stress, autophagy is insufficient to maintain homeostasis and thus autophagy failure is associated with programmed cell death. Programmed cell death can be initiated by a number of extrinsic and intrinsic factors in cells, including activation of death receptors, membrane damage or stress experienced by intracellular organelles including mitochondria, the nucleus, the endoplasmic reticulum and lysosomes^31–34^. The Nomenclature Committee on Cell Death has recently updated their guidelines for the classification of regulated cell death based on current knowledge of key signal transduction pathways and pathophysiological outcomes of the process. No fewer than 12 different subtypes of regulated cell death are proposed and only three of these are fully dependent on caspase activation. Intrinsic and extrinsic apoptosis rely on executioner caspase activation whereas pyroptosis relies on inflammatory caspase activation. The remaining nine subtypes of regulated cell death are forms of caspase independent cell death and can occur in the absence of caspase activity; autophagy-dependent cell death, entotic cell death, ferroptosis, immunogenic cell death, lysosome-dependent cell death, mitochondrial permeability transition-driven necrosis, necroptosis, NETotic cell death, and parthanatos^35^.

Energy-independent modes of cell death such as necrosis are typically described by morphological features for example loss of membrane integrity, and releasing cellular contents into the cytosol^36^ which in turn initiates an immune response, resulting in the cell being phagocytosed. However, a form of necrosis that is energy-dependent and regulated in cells has been discovered and is known as necroptosis, suggesting that most forms of physiological cell death are energy dependent involving intracellular and sometimes extracellular signal transduction.

We previously identified a ROS-independent mechanism of cell death in U373MG GBM cells that was dose-dependent and independent of both JNK and caspase activation^12^. U373MG GBM cells are known to have a very high tolerance to ROS such as H_2_O_2_ owing in part to expressing mutated p53^37^. The current study aims to investigate further the triggers involved in activation of CICD and the biochemical alterations evident when ROS-resistant GBM cells are exposed to toxic doses of plasma. We find evidence that CICD is preceded by rapid accumulation of acidic vesicles in cells, but no biochemical features of autophagy are evident. Instead, a large proliferation of lysosomes in cells is associated with cell death following plasma treatment.

## Results

### CAP results in rapidly induced cytotoxicity

**Figure 1:**
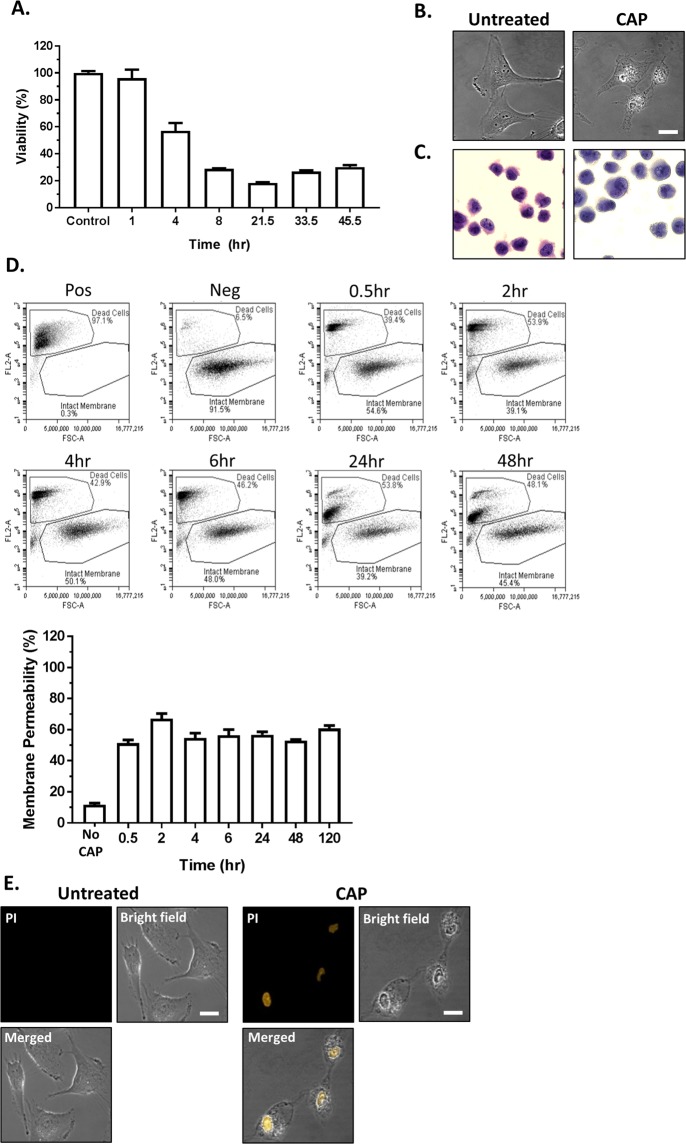
CAP induces rapid cytotoxicity in GBM cells. (**A**) U373MG cells were exposed to CAP at 75 kV for 180 seconds and analysed by Alamar blue cell viability assay at 1, 4, 8, 21.5, 33.5, 45.5 hours post CAP treatment. Each time point was normalised to an untreated control that was left under the same conditions as the CAP-treated plate. Data shown was normalised to the untreated control and is shown as the % mean ± S.E.M. (*n* = *minimum 12*). (**B**) U373MG cells were treated with CAP for 60 sec. Images were taken using confocal microscopy 4 hours post treated of both treated and untreated samples to determine morphological differences. Scale bar is 10 µm (**C**) Cells were subjected to CAP for 180 seconds, and fixed and stained with H&E 24 hours post treatment. Cells were examined under light microscopy and images were captured. (**D**) Verification of apoptotic nuclear membrane degradation was measured up to 0–120 hr after CAP exposure (75 kV for 60 sec) by flow cytometry using propidium iodide (10 µg/ml). Data shown was normalised to the untreated control and is shown as the % mean ± S.E.M. (*n* = 4). **(E)** *In-situ* verification of nuclear membrane degradation was measured 4 hr after CAP exposure (75 kV for 60 sec) by confocal microscopy using propidium iodide (10 μg/ml).

Previously we have demonstrated dose-dependent cytotoxicity of CAP in U373MG GBM cells^12^. Figure 1A depicts representative kinetic cytotoxicity results obtained when GBM cells are exposed to CAP for 180 seconds. Inhibition of normal metabolic processes in GBM cells by CAP treatment was almost immediate. It was observed that there was a significant reduction in cell viability 4 hours post CAP treatment compared to untreated controls, with an ~80% loss in cell viability after 24 hours. Gross morphological changes were detected in cells 4 hours after CAP treatment, including the apparent presence of internal vesicles (Fig. 1B). Morphological assessment of cell death was carried out using H&E staining 24 hours after treatment (Fig. 1C). No evidence of any morphological hallmarks of apoptosis were observed, specifically, no evidence of any increase in membrane apoptotic blebbing, nuclear condensation/fragmentation or apoptotic body formation in the CAP-treated cells. Similarly, we did not find any morphological evidence of necrosis. However, multiple internal vesicles were observed in CAP-treated cells that were not present in untreated controls.

Propidium iodide (PI) was added to the cells post CAP treatment and analysed by flow cytometry. Figure 1D demonstrates the uptake of PI, which has bound to the nucleic acids within the nucleus of CAP-treated GBM cells and compared to the untreated control. A rapid increase in membrane permeability was observed (Fig. 1D *P < 0.05). Interestingly, at 24 and 48 hours a second population is observed when compared to early 6 hr time points. The rapid uptake of PI indicates membrane damage but when compared with the Alamar blue data (Fig. 1A) and morphological data (Fig. 1C), our data suggest we are measuring membrane permeabilisation and damage induced by CAP that precedes biochemical and morphological indicators of cell death.

This was further confirmed by confocal microscopy, Fig. 1E, which demonstrates localisation of PI, which has bound to the nucleic acids within the nucleus of CAP-treated GBM cells. In contrast, no fluorescent signal was observed in the untreated control cells, indicating the presence of a compromised plasma membrane in CAP treated cells. Fluorescence was quantified using ImageJ software and compared to the untreated control. The increase in fluorescence was quantified and a significant (*P < 0.05) increase in fluorescence following CAP treatment was observed (data not shown).

### CAP induces ROS and caspase-independent cell death in GBM cells

**Figure 2:**
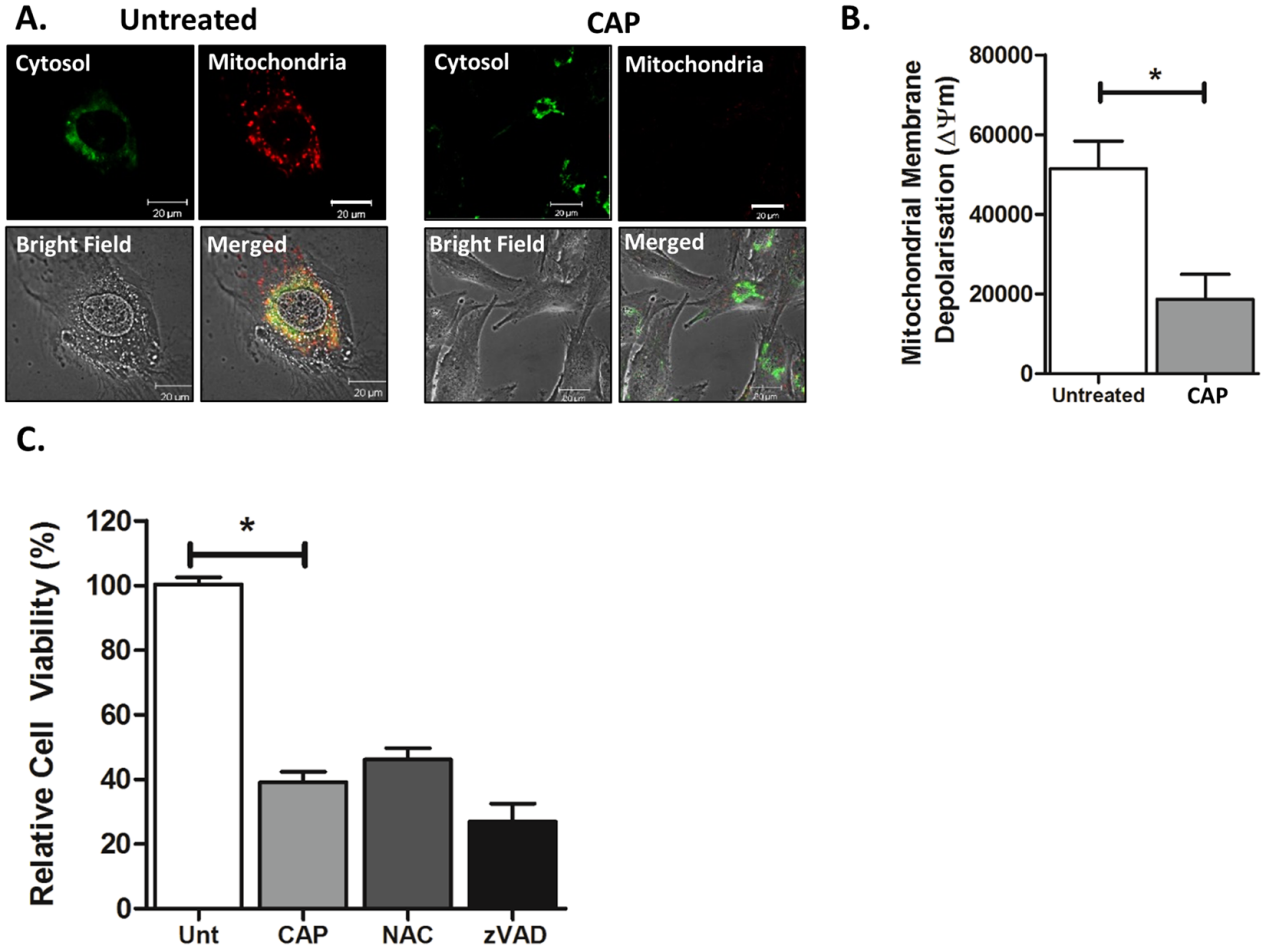
CAP induces ROS- and caspase-independent cell death in GBM cells. (**A**) *In-situ* verification of ΔΨm was measured 4 hrs after CAP exposure (75 kV for 60 sec) by confocal microscopy using JC-1 2.5 µg/ml (**B**) The level of fluorescence was quantified using ImageJ software and compared to the untreated control. Statistical analysis was carried out using an unpaired t-test (*P < 0.05). (**C**) U373MG cells were preloaded for 1 hr with inhibitors 4 mM NAC or 50 µM zVAD-FMK prior to CAP treatment (75 kV for 60 sec). After 48 hours, cells were analysed using the Alamar blue assay. Data shown was normalised to the untreated control and is shown as the % mean ± S.E.M. (*n* = *minimum 20*). Statistical analysis was carried out using One-Way ANOVA with Bonferroni’s post-test. (*P < 0.05).

Mitochondria play a key role in programmed cell death and autophagy^38^. Oxidative stress, such as reactive oxygen species generated from CAP, targets the mitochondria which can result in mitochondrial dysfunction and therefore cell death, but similarly up regulation of the autophagy process can result in mitochondrial dysfunction and cell death^39,40^. Previous studies have demonstrated that CAP treatment results in loss of the mitochondrial membrane potential (ΔΨm)^12,41^. ΔΨm was examined using molecular fluorescent probe JC-1 by confocal microscopy. As demonstrated in Fig. 2A, JC-1 aggregates in healthy mitochondria exhibiting punctated red fluorescence. In contrast there is a significant reduction in JC-1 aggregates (red fluorescence) where the membrane potential has decreased as a result of CAP treatment. Quantification of the fluorescence intensity demonstrated a significant decrease (p < 0.05) in the red fluorescent signal between CAP-treated cells and untreated healthy controls (Fig. 2B). ROS-dependent activation of caspases and apoptosis are believed to be the primary routes of cell death in cancer cells exposed to CAP. However, Fig. 2C shows that U373MG cells loaded with antioxidant N-acetyl cysteine or caspase inhibitor zVAD-FMK offer no protection when treated with CAP, in agreement with our previous data^12^. Together, our results indicate that ROS-dependent activation of apoptosis is not necessary for cell death when U373MG GBM cells are exposed to CAP.

### 3-MA inhibits cell death in response to CAP but not the formation of AVOs

**Figure 3:**
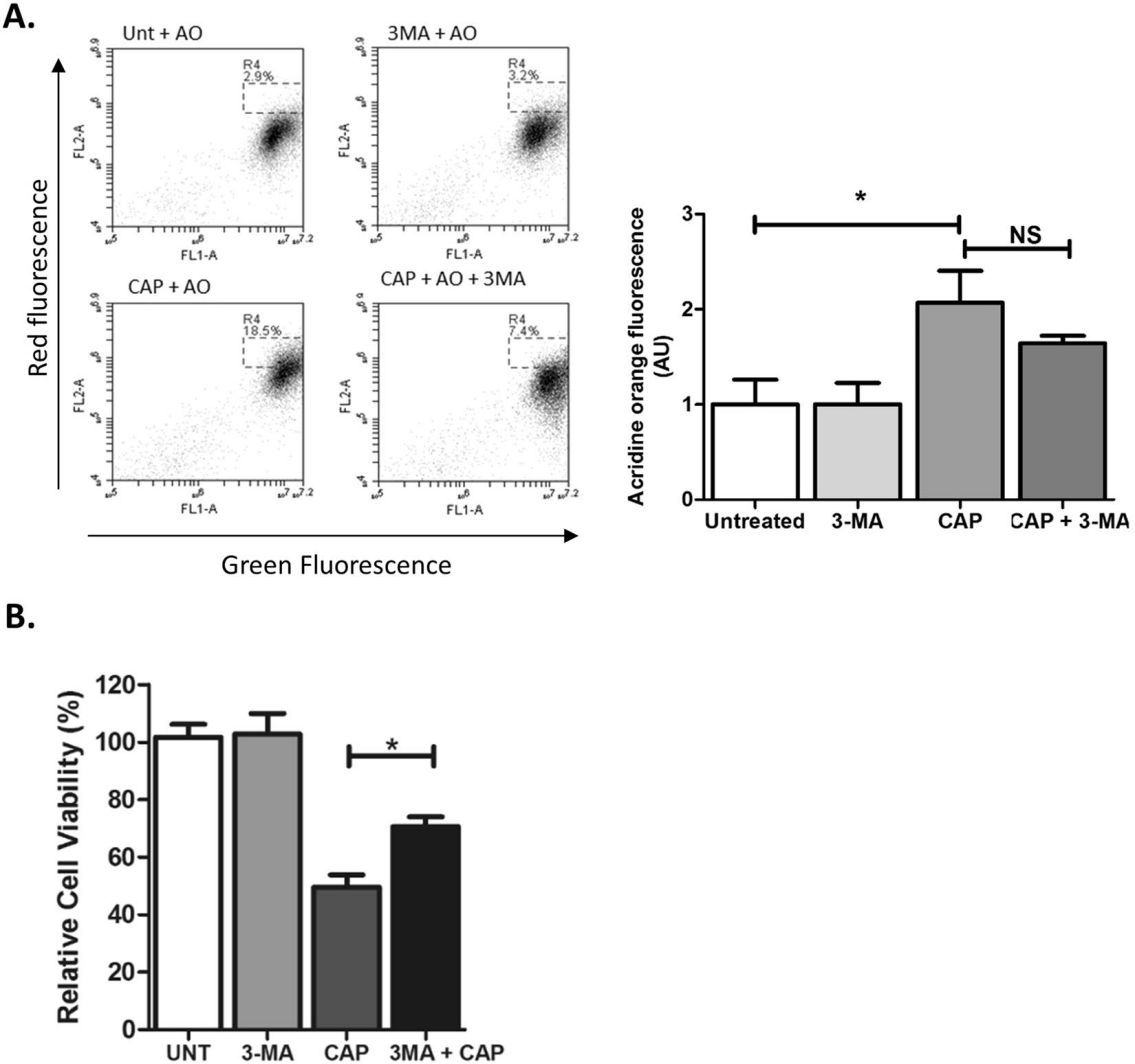
CAP induces the formation of acidic vesicles organelles (AVO’s). (**A**) U373MG cells were preloaded for 1 hr with 5 mM 3-MA. Cells were exposed to CAP for 60 sec at 75 kV. After 48 hours cells were analysed using the Alamar blue assay. Data shown was normalised to the untreated control and is shown as the % mean ± S.E.M. (*n* = *minimum 20*). Statistical analysis was carried out using one-way ANOVA (*P < 0.05). (**B**) U373MG cells were pre-treated with or without 5 mM 3-MA for 1 hour then exposed to CAP at 75 kV for 60 seconds. After a 48 hr incubation period cells were loaded with 1 µg/ml AO dye and analysed by flow cytometry. Data shown depicts the formation of AVO’s by quantitative shifts the FL2 channel, red fluorescence intensity ratio in both treated and untreated samples in the presence or absence of the 3-MA. (1. 3-MA, 2. Untreated, 3. CAP, 4. CAP & 3-MA). Data was quantified using the mean fluorescence index and normalised to the untreated control. Statistical analysis was carried out using one-way ANOVA with Bonferroni’s post-test (*p < 0.05).

In consideration of the rapid appearance of intracellular vesicles, and observations by other research groups, we postulated that autophagy might be up-regulated following CAP treatment and thus an early indicator of cytotoxic insult and resultant cell death. The observed accumulation of intracellular vesicles (Fig. 1B,C) supported this hypothesis. Cells were loaded with acridine orange (AO), a cell-permeable, nucleic acid-selective fluorescent cationic dye and analysed by confocal microscopy. AVOs were observed in CAP treated cells and were significantly lower in untreated cells (*P < 0.001, data not shown). This was confirmed and quantified using flow cytometry 48 hours post treatment with CAP. Figure 3A demonstrates an increase in AVO formation as evidenced by the increase in FL2 red fluorescence signal compared with the untreated cells. When quantified, a significant difference in the mean fluorescence index was observed between treated and untreated cells (P < 0.05, Fig. 3A). The presence of red fluorescence from AO staining validates the formation of AVOs, which are a significant characteristic of the autophagic process. The formation of AVOs following CAP treatment was also observed in HeLa and A459 cells (see Supplemental Fig. 6).

Interestingly, 48 hours following CAP treatment when co-treated with 3-MA, there was no evidence of a reduction in AVO formation (Fig. 3A). This was also confirmed for cells analysed 4 hours post CAP treatment (data not shown). When quantified, there was no observed decrease in the mean fluorescence index of FL2 (red fluorescence) in the presence of 3-MA. The FL2 mean fluorescence index was quantified, and statistical analysis confirmed that 3-MA does not yield any significant difference in the formation of AVOs following treatment with CAP. Following confirmation of AVO formation, it was hypothesised that CAP treatment results in activation of the autophagic pathway. As demonstrated in Fig. 3B, using spectrophotometric analysis, the autophagy inhibitor 3-methyladenenine (3-MA) significantly alleviates the cytotoxic effect of CAP over a 48-hour period.

### CAP treatment has no effect on mTORC1 signalling or the autophagic pathway

3-MA inhibits PI-3K activity, which generates the phospholipid PtdIns3P, a critical signal for activation of autophagy. To investigate whether autophagy signalling was initiated, we assessed Atg4-dependent processing and lipidation of LC3B, converting it from LC3B-I to LC3B-II. LC3B processing is a reliable marker for induction and formation of autophagosomes. LC3B-II can be detected by SDS-PAGE and western immunoblotting^42^.

**Figure 4:**
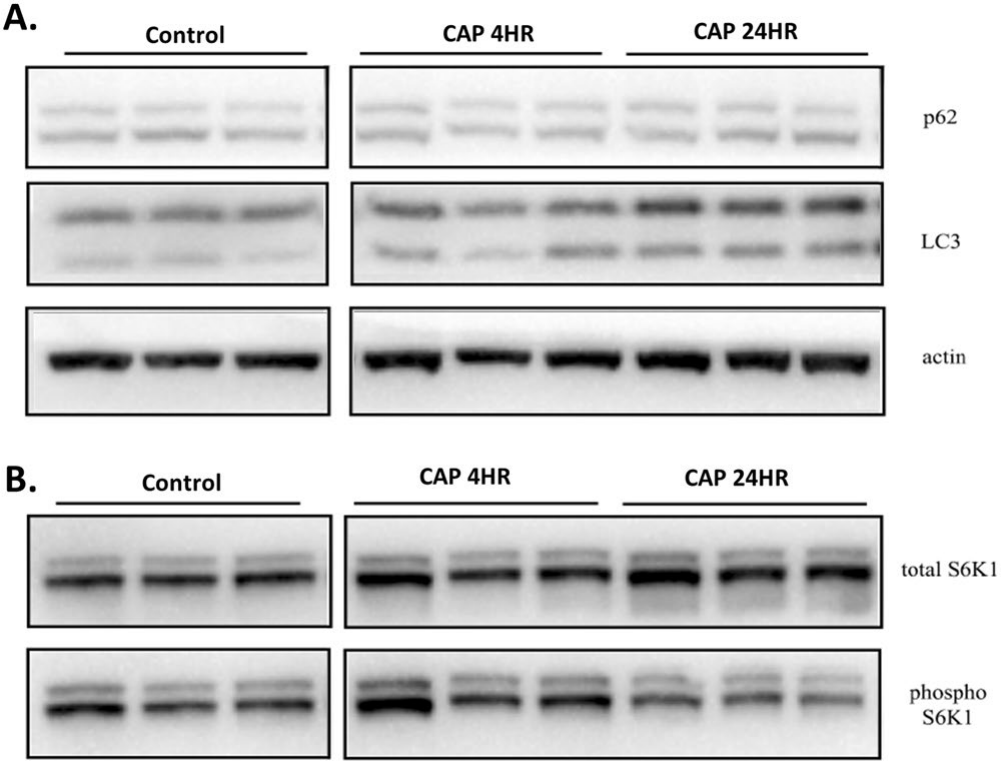
Autophagic markers are not activated by CAP. (**A**) Cell extracts were resolved by 10% SDS-PAGE and proteins electroblotted to PVDF membrane. Membranes were probed with antibodies that recognise total and pThr389 S6K1 or LC3B and p62/SQSTM1. Actin was used as a loading control. The antibody-labelled proteins were detected by Enhanced Chemi-Luminescence (ECL). Blots have been cropped for clarity and conciseness. Full-length blots and densitometry data are presented in Supplementary Fig. 4.

We observed no increase in LC3B-II 4 hours after treatment with CAP and no evidence of LC3B-I (upper band) to LC3B-II (lower band) conversion (Fig. 4A). This was further substantiated by the lack of any change in p62/SQSTM1 expression, a ubiquitin-binding autophagic adaptor protein which is degraded when autophagy is activated^43^. Expression of p62 after 4 hours post CAP treatment was similar to the control (Fig. 4D). When LC3B-II and p62 expression was analysed at 24 hours, accumulation of neither LC3B-II nor p62 was evident. With no change in P62 expression levels evident and no processing of LC3B, our data demonstrates (Fig. 4A) that autophagy is not activated following CAP treatment.

Control of autophagy is exerted through the PI3K/Akt/mTORC1 signalling pathway, whereby mTORC1 functions as a master regulator of biomass production. Phosphorylation of S6K1 at Thr389 is a hallmark of mTORC1 activation, and therefore correlates with inhibition of autophagy in most situations. To further confirm our findings, we investigated the status of threonine 389 phosphorylation on S6K1 following incubation with CAP. We observed no reduction of phospho-Thr389 on S6K1 at 4 hours despite the presence of AVOs (data not shown). We did observe a significant decrease after 24 hours but this is likely to be a consequence of cells undergoing cell death (Fig. 4B and Supplementary Material 4). Together, it can be inferred from our data that CAP does not alter mTORC1 signalling or activity of the autophagy pathway, therefore the acidic vesicle organelles that have been identified by acridine orange are mostly other acidic compartments such as lysosomes.

### CAP stimulates the accumulation of lysosomes

**Figure 5:**
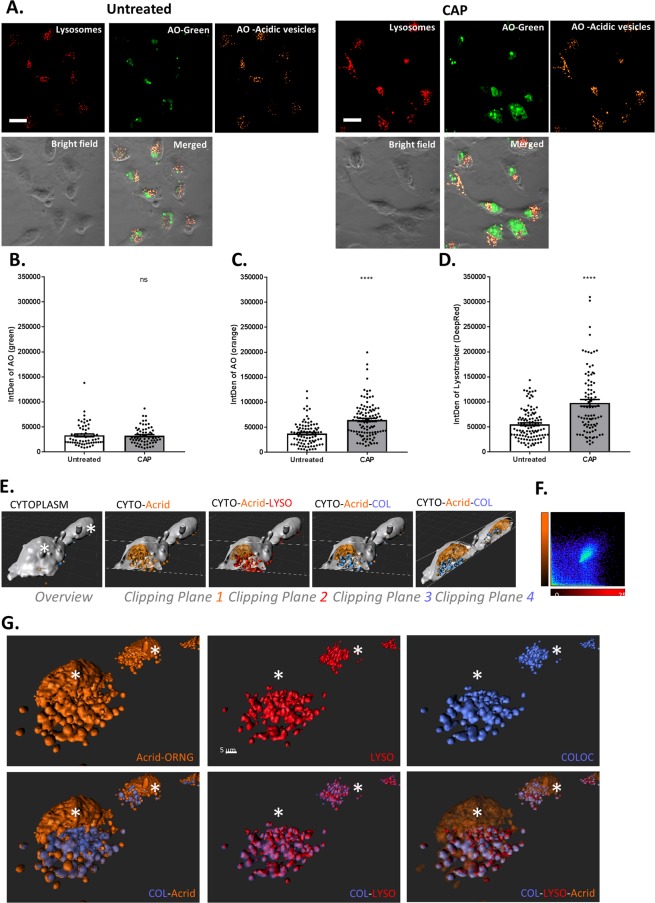
Acidic vesicles co-localize with lysosomes. (**A**) *In-situ* verification of lysosomal formation was measured 24 hr after CAP exposure (75 kV for 60 sec) by confocal microscopy using LysoTracker™ Deep Red (50 nM) and acridine orange (1 µg/ml). (**B**–**D**) The fluorescence intensity of AO green and orange channel, and LysoTracker Deep Red was quantified using ImageJ software and compared to the untreated control. Statistical analysis was carried out using an unpaired t-test *(n* = *111)* (*P < 0.001). The scale bar is 10 µm. (**E**) Two representative cells (indicated by asterisks [*]) imaged with confocal microscope were analysed with 3D rendering software. We visualized the cytoplasm (grey) the AO-stained vesicles and nucleus (orange) and lysosomes (red). The co-localisation of orange and red is indicated with a blue isosurface. We display different images with oriented clipping planes to observe the localization of vesicles. (**F**) Plot showing the co-localisation analysis of Lysotracker™ Deep Red and AO channels within the confocal scan. (**G**) Top panels show the detailed analysis of the co-localisation of acidic vesicles (orange) and lysosomes (red) in the analysed cells. Co-localising vesicles are indicated in blue isosurfaces. The bottom panel demonstrates colocalising vesicles by rendered transparencies of the isosurfaces.

Having demonstrated that CAP is not inducing autophagy, it was postulated as to whether the acidic vesicles being formed may be an influx of lysosomes. Lysosomes are membrane-bound organelles that contain hydrolytic enzymes which function to recycle damaged organelles. They are both structurally and physiologically similar to autophagosomes. Additionally, they are equally as harmful to the cell, inducing lysosomal membrane permeabilisation (LMP) following damage to the lysosomal membrane. Following CAP treatment, cells were stained with the lysosomal marker LysoTracker™ Deep Red and AO which labels all acidic organelles including lysosomes and were examined by confocal microscopy. The red fluorescence demonstrates cells stained with LysoTracker™ Deep Red and therefore stained lysosomes. The green fluorescence indicates AO binding to DNA, and the orange fluorescence was AO in the acidic vesicles. It was determined from the manufacturing emission profile that Deep Red has low emissions in the orange channel, therefore demonstrating the fluorescence from AO is excluded from the red channel and is only demonstrated in the orange channel. In addition, Fig. 5A, confirms that there is no co-localisation between the green and orange channels. This data (Fig. 5A) exhibits a significant (*P < 0.001) increase in the presence of lysosomes following CAP treatment compared to the untreated control. To further distinguish between AO-stained vesicles and to further confirm the presence of lysosomes, acidic vesicles were co-localised with LysoTracker™ Deep Red. 3D isosurface rendered z-stack confocal images were generated (Fig. 5B). The co-localisation of AO and LysoTracker™ Deep Red is depicted in blue, as demonstrated by the co-localisation plot (Fig. 5C). It is noted that AO can also act as a nuclear stain^44^. It binds to DNA and double-stranded RNA and emits green fluorescence, whereas it binds with single-stranded DNA and RNA to emit orange fluorescence. As seen in Fig. 5D (marked with a white asterisk) AO has bound to the nucleus of the cell emitting orange fluorescence demonstrating the presence of single stranded DNA and or RNA. As seen in the final panel of Fig. 5D, there is significant overlap between AO and LysoTracker™ Deep Red, thus confirming that AO was in fact staining lysosomes.

## Discussion

This study demonstrates that the cytotoxic insult induced by 75 kV CAP for 180 seconds occurs rapidly, with 50% cell death observed after only 5 hours and less than 20% viable after 24 hours. Additionally, shorter doses of CAP induce rapid cell death, but the magnitude of cell death was lower (i.e. CAP treatment for 60 seconds resulted in 40–50% viability after 24 hours). Our findings that ROS and Caspase inhibitors do not affect cell death was confirmed by morphological assessment of cells using H&E, where we observed no evidence of classical morphological indicators of either apoptosis or necrosis^45^. This correlates with recently published data using CAP in U373MG cells, where a significant increase in cytotoxicity after 24 hours using a different indicator of cell death (MTT) was reported^18^. Siu *et al*. also suggested that cell death of U373MG in response to helium jet CAP is a result of apoptosis or necrosis depending on treatment time^18^. The authors observed a loss in cell viability within 24 hours, however only a small increase in caspase 3/7 activity was observed at 48 hours and not at earlier time-points, indicating that caspases may not play an important role. Recent recommendations on detection of apoptosis suggest a combinational approach; the use of inhibitor studies, combined with biochemical criteria and corroboration using morphological data^46^. We have combined inhibitors with biochemical assays and morphological assessment to identify mitochondrial dysfunction and loss of plasma membrane integrity which are features of cell death induced by CAP. However, caspase activation and hallmark morphological features of apoptosis are not apparent at either 60 seconds or 180 seconds, suggesting that caspase-dependent apoptosis is not playing a role in our model.

Caspase-independent cell death has previously been associated with autophagy and macroautophagosome formation^47^. There is a very close relationship between the autophagic process and apoptosis^25,27^, with some studies suggesting that autophagy may coordinate with apoptosis to induce cell death^48^. Similarly, it has previously been suggested that the mitochondria maybe an organelle that integrates and regulates both processes^49^. Similar to that described by others, our previous study confirmed depolarisation of the mitochondrial membrane^12,50^. Here it is identified that CAP in fact results in a rapid depolarisation of mitochondrial membrane potential, as quickly as 4 hours post CAP treatment. The kinetics of mitochondrial depolarisation is indicative of a response that does not require *de nova* gene expression. It has recently been demonstrated that both prostate cancer and normal cells exposed to CAP for longer (180 and 600 s) treatment times activated autophagy as a cytoprotective mechanism in an attempt to cope with overwhelming stress^20^. A distinct morphological feature of autophagy is the formation of acidic vesicular organelles (AVOs)^51^. This study reports the detection of AVOs by both flow cytometry and confocal microscopy as early as 4 hours post CAP treatment. However, while often used as an indicator of autophagy, AO is an acidic dye that can permeate cell membranes of other vesicles that maintain a low pH, including lysosomes and endosomes. Therefore, this method can only be used to detect the presence of AVOs and cannot confirm activation of autophagy as a standalone assay. To further examine the possibility that CAP activated autophagy, we employed the autophagy inhibitor 3-MA^52^, where partial protection against the cytotoxic effects of CAP was observed. The PI3K/Akt/mTORC1 pathway is over-activated in many cancer cells. PI3K class-I inhibits autophagy initiation by activating mTORC1, whereas PI3K class-III generates PtdIns(3)P for autophagosome nucleation during autophagy. 3-MA is considered a selective inhibitor of Class III PI3K activity which is necessary for autophagy, however, long- term exposure to 3-MA can also induce autophagy^53^ by inhibiting class I PI3K activity^54,55^. Interestingly, and in contrast to our cell viability data, when combined with flow cytometry, we did not observe any significant reduction in the formation of AVO’s after 4 or 48 hours, therefore, we postulated that presence of AVO’s following CAP treatment may be an acidic vesicle such as lysosomes or endosomes. Lysosomes are membrane bound organelles that contain hydrolytic enzymes such as cathepsins and can degrade both intracellular and extracellular material. In recent years it has become accepted that lysosomes and cathepsins have other functions other than intracellular degradation and recycling^56^. Lysosomal membrane permeabilization which is carried out by the cathepsin proteases can have necrotic, apoptotic or apoptosis-like features depending on the extent of the leakage and the cellular context^56–58^. Interestingly, it has been shown that when cells are exposed to oxidative stress, this can result in peroxidation of the lysosomal membrane, thus the lysosome becomes leaky. If the extent of the damage (i.e. the leak in the membrane) is moderate the cell may survive following activation of autophagy, however, if the damage is more extensive the cells die^59^. Co-localization studies with AO and LysoTracker™ Deep Red and 3-D modelling identify that CAP-stimulated AVO’s are most likely lysosomes. Lysosome accumulation following plasma treatment is a cellular change that will be useful in developing future programmable therapeutics using prodrugs and nanomaterial strategies to amplify the cytotoxicity in a targeted fashion. It is thought that the proliferation of lysosomes following CAP treatment are the prelude to autophagy, prior to the fusion with autophagosomes, however, our data demonstrates no accumulation of LC3B-II or p62 indicting that autophagy is not activated following CAP treatment. As evident from the 3D-modeling images, the staining indicts that the lysosomes are surrounding the nucleus. It has been previously noted in *Drosophila* that lysosomes become highly abundant in apoptotic cells, enabling the latter to be distinguished from non-apoptotic cells, more interestingly it was noted that lysosomes surround the apoptotic nucleus, which become the target for the release of lysosomal hydrolytic enzymes into the nucleus, initiating terminal genome destruction^44^.

In recent years, it has been demonstrated that lysosome permeabilization, has been shown to initiate a cell death pathway^60,61^ similarly to that observed with mitochondrial outer membrane permeabilization^12,62^. Cathepsins which are contained in the lysosome can be released into the cytosol and initiate the lysosomal pathway of apoptosis through the cleavage of Bid and the degradation of the anti-apoptotic Bcl-2 homologues^44^. More interestingly, lysosomal membrane permeabilization (LMP) mediated cell death is induced by reactive oxygen species (ROS). In an environment where the presence of oxidative stress is abundant, ROS can pass through the lysosomal membrane and, in the presence of free iron, catalyse Fenton reactions to produce highly toxic intermediates that damage lysosomal proteins, such as Hsp70^50^.

In conclusion this study has confirmed that autophagy is not involved in CAP-stimulated cell death. Instead, membrane damage is an early feature and is rapidly followed by morphological and biochemical indicators of cell death. We have demonstrated proliferation and accumulation of peri-nuclear lysosomal vesicles following CAP treatment, likely linked to membrane damage response, and 3-MA-dependent cell death, thereby providing evidence that the endocytic/lysosomal pathway may be complicit in cell death. However, further work is needed to fully elucidate whether CAP treatment stimulates and triggers LMP-associated cell death.

## Materials and Methods

### Cell culture

Human glioblastoma (U373MG-CD14) cells were obtained from Dr Michael Carty (Trinity College Dublin). U373MG cells, were cultured in DMEM/F12 (Sigma-Aldrich, Arklow, Ireland) supplemented with 10% FBS (Sigma-Aldrich, Arklow, Ireland) which were maintained in a humidified incubator containing 5% CO_2_ at 37 °C. Media was changed every 2–3 days until 80% confluency was reached. Cells were routinely sub-cultured using a final 1:1 ratio of 0.25% trypsin (Sigma-Aldrich, Arklow, Ireland) and 0.1% EDTA (Sigma-Aldrich, Arklow, Ireland).

### Cold atmospheric plasma device

The DIT 120 prototype systems consists of a high voltage transformer (with input voltage 230 V at 50 Hz), a voltage variac (0–100%, output voltage could be controlled within 0~120 kV). There are two 15-cm diameter aluminium electrodes for generation of plasma that were separated by a 1 mm thick polypropylene sheet which served both as a sample holder and as a dielectric barrier with a thickness of 1–2 mm^63^. The distance between the electrodes was altered depending on the culture plate used (14.2 mm–26.6). Voltage and input current characteristics were monitored using the InfiniVision 2000X-Series Oscilloscope (Agilent Technologies Inc., Santa Clara, CA, USA). The atmospheric air condition at the time of treatment was 45% relative humidity (RH) and 22 °C. All samples were exposed to 75 kV and depending on the experiment being carried out the exposure time was altered (0–180 sec). Previous characterisation studies carried out on the DIT 120 demonstrate inhibition of ROS induced cytotoxicity following the addition of antioxidants such as pyruvate (commonly found in cell culture media) (Supplementary Fig. 7). Studying the effects of CAP in a system with high levels of anti-oxidant capacity offers a better representation of the effects likely observed *in situ*^64,65^.

### Alamar blue cell viability assay

U373MG cells were seeded at a density of 1 × 10^4^ per well into 96- well plates (Sigma-Aldrich, Arklow, Ireland) and allowed to adhere and grow overnight. Media was removed for the duration of CAP treatment and fresh media was replaced immediately after treatment and incubated at 37 °C as indicated. No deleterious effects were observed in the vehicle control samples. Cell viability was analysed using Alamar Blue (Fischer Scientific, Ballycoolin, Ireland). Cells were washed once with sterile PBS, incubated for 2.5 hours at 37 °C with a 10% Alamar blue solution. Fluorescence was measured using an excitation wavelength of 530 nm and emission wavelength of 595 nm on a Victor 3 V 1420 (Perkin Elmer) multi-plate reader.

### Haematoxylin and Eosin staining

Cells were plated in T25 flask and incubated overnight as above, cells were treated with CAP as described above or left untreated. Cell were harvested, the pellet was fixed using 70% ethanol (Sigma Aldrich, Arklow, Ireland). A monolayer of both treated and untreated of cells was generated using a cytospin. Cells were then stained with Harris Haematoxylin for two minutes and 1% Eosin for 3 minutes. Cells were dehydrated in alcohol and xylene and analysed by light microscopy (Olympus Bx41).

### Confocal microscopy

U373MG cells were plated in 35 mm glass bottom dishes (MatTek Corporation, USA) at 1 × 10^4^ cells per dish and incubated for 24 hrs. Media was removed and cells were exposed to plasma for 60 sec at 75 kV after which fresh media was added. 4 hours after treatment cells were loaded with AO (1 µg/ml for 15 minutes 37 °C) or JC-1 mitochondrial membrane potential indicator (2.5 µg/ml for 30 minutes 37 °C), LysoTracker™ Deep Red (Thermo Fisher Scientific) and images were captured on a Zeiss 510 LZSM confocal inverted microscope. JC-1 was detected by a multi-track dual excitation and emission scan, namely 488 nm excitation and JC-1 emission between 505–530 nm with a band-pass filter and secondly 543 nm excitation and JC-1 emission above 560 nm with a 560 nm long-pass filter. AO was also detected by a multi-track dual excitation and emission scan, namely 477 nm excitation and AO emission above 585 nm with the aid of a long-pass 585 nm filter and secondly by 488 nm excitation and the AO emission between 505–530 nm with a band-pass filter. LysoTracker™ Deep Red was also detected by a multi-track dual excitation and emission scan, namely 633 nm excitation and LysoTracker™ Deep Red emission above 635 nm with the aid of a long-pass 635 nm filter. All images were recorded live through PBS. The total cell fluorescence was calculated using ImageJ (v1.49, NIH) software. An outline was drawn around each individual cell, as previously described^15^. The total corrected fluorescence was then calculated as follows; Total corrected cell fluorescence (TCCF) = Integrated density − (area of selected cell × mean fluorescence of background readings)^15^.

### Inhibitor studies

U373MG cells were pretreated with NAC (4 mM), 3-methyladenine (3-MA) (5 mM) (Sigma Aldrich, Arklow, Ireland), zVAD-FMK (50 µM) (InvivoGen, Toulouse, France) for 1 hour prior to plasma treatment, and was added again immediately after CAP treatment. Cells were treated for 180 sec at 75 kV. Cell viability was assessed 48 hours later using Alamar Blue. Please see Supplemental Fig. 8 for zVAD-FMK optimisation and dose- response curves.

### Flow cytometry

#### Propidium iodide

Live and dead staining cells were demonstrated using propidium iodide (PI) (Sigma Aldrich, St. Louis, USA). U373MG cells were seeded in 6-well plate at 2.5 × 10^5^ cells per well and treated with CAP 75 kV, for 60 s. Cells were harvested at different time points post-treatment to CAP (0.5h, 2h, 4h, 6h, 24h, 48h and 120h) and were centrifuged to form a pellet. The pellet was resuspended in 1 ml PBS and was stained with 10 µg/ml PI for 1 minute. Live and dead cells were analysed with BD Accuri C6 flow cytometer (BD, Oxford, UK), FL3 vs FSC which detects PI fluorescence, where PI binds to nuclear degradation from dead cells.

#### Acridine orange

Cells were seeded in 6-well plates; cells were then treated with CAP as described above for 60 seconds, and or 3-MA (5 mM) and further incubated for 48 hours. Cells were harvested both 4 hours and 48 hours post treatment and stained with 1 µg/ml acridine orange (Sigma Aldrich, Arklow Ireland) incubated at 37 °C for 20 minutes, washed twice with sterile PBS and analysed by flow cytometry (BD Accuri C6). Detection of AVO’s by acridine orange was performed using the FL1 (green) vs FL2 (orange) channels and compensation was set at approximately at 7% removing FL2 signal from FL1 and approximately 16% removing FL1 signal from FL2 for each plot.

### Western immunoblotting

Cells were washed once with ice-cold PBS, then lysed in lysis buffer (50 mM Tris-Cl, pH 7.5, 1 mM EGTA, 1 mM EDTA, 0.3% (w/v) CHAPs, 1 mM sodium orthovanadate, 50 mM sodium fluoride, 5 mM sodium pyrophosphate, 0.27 M sucrose, 0.1% (v/v) 2-mercaptoethanol) containing complete™ Protease Inhibitor Cocktail, (Roche Diagnostics, West Sussex, UK)). Protein concentrations were determined by Coomassie (Bradford) Protein Assay, (Thermo-Pierce, Northumberland, UK) against a BSA standard curve. For Western immunoblotting of whole cell extracts (WCL), samples were prepared by adding 4x LDS buffer directly to cell lysates, sonicated to disrupt genomic DNA and heated for 10 mins at 70 °C. Protein extracts were resolved by 10% Bis-Tris SDS-PAGE and gels were transferred to Immobilon-P PVDF membrane, then blocked in 50 mM Tris-Cl, pH 7.5, 150 mM NaCl and 0.2% Tween20 (TBS-T) containing 5% skimmed milk (block solution). Membranes were washed three times in TBS-T before probing with the indicated primary antibodies (anti-LC3B, anti-p62/SQSTM1, anti-S6K1 and anti-pThr389-S6K1 were from Cell Signalling Technology, and anti-actin was from Sigma-Aldrich), diluted in block solution for either 2 hours at ambient temperature or overnight at 4 °C. Membranes were again washed three times in TBS-T, before incubation with species-specific horseradish peroxidase (HRP)-conjugated secondary antibody for 1 hour at ambient temperature. Membranes were washed three more times in TBS-T before antibody-labelled proteins were detected by Enhanced Chemi-Luminescence (ECL) (Millipore) using a digital gel documentation system (Bio-Rad). Blots have been cropped for clarity and conciseness. Full-length blots are presented in Supplementary Material 4.

### Isosurface rendering

Confocal images were deconvolved using Huygens Professional image processing software package (Scientific Volume Imaging b.v., Hilversum, The Netherlands) or AutoQuant X3 image deconvolution software (Bitplane AG, Zurich, Switzerland). Once images were processed to maximize quality, the colocalisation of the fluorescent materials was measured in voxels using Imaris 8.1 image visualization and analysis software (Bitplane AG, Zurich, Switzerland). Then, the colocalising voxels between the different fluorophores were extracted in a new channel to visualize the colocalisation. 3D isosurfaces were generated with the adequate detail resolution threshold and shadowing. Finally, 3D rotations were generated, and clipping planes were applied to show the position of cellular structures of interest, including the relevant colocalising material.

### Statistical analysis

All experiments were performed at least three independent times with a minimum of five replicates per experiment. Data shown is pooled and presented as mean ± SEM (n = total number of replicates) unless stated otherwise. Statistical analysis, and curve fitting was performed using Prism 5, GraphPad Software, Inc. (USA). Using Shapiro-Wilk normality test to calculate normality, our data was then normalised to the untreated control. Unless otherwise indicated differences were considered significant with a *P value < 0.05.

## Supplementary information

Supplemental Material

## Data Availability

All datasets can be viewed in tabular form in Supplementary Information.
