# Supplementary material for: Cold Atmospheric Plasma induces accumulation of lysosomes and caspase-independent cell death in U373MG glioblastoma multiforme cells
Source: Sci Rep. 2019 Sep 9;9:12891. doi: 10.1038/s41598-019-49013-3 (PMC6733837; doi:10.1038/s41598-019-49013-3)

**Cold Atmospheric Plasma induces accumulation of lysosomes and caspase-independent cell death in U373MG Glioblastoma multiforme cells**

**Gillian E Conway^1-3,6^, Zhonglei He^1-3^, Ana Lacramioara Hutanu^7^, George Paul Cribaro^8^, Eline Manaloto^1-3^, Alan Casey^2,4^, Damien Traynor^2^, Vladimir Milosavljevic^2,4^ Orla Howe^2,3,5^, Carlos Barcia^8^, James T. Murray^7^, Patrick J Cullen^1- 3, 9^, James F Curtin^1-3^**

^1^School of Food Science & Environmental Health, ^2^FOCAS Research Institute, ^3^Environmental Sustainability & Health Institute, ^4^School of Physics & Clinical & Optometric Sciences and ^5^School of Biological & Health Sciences, Technological University Dublin, Ireland; ^6^*In-Vitro* Toxicology Group, Institute of Life Science, Swansea University Medical School, Swansea University, Singleton Park, Swansea ^7^School of Biochemistry & Immunology, Trinity Biomedical Sciences Institute, Trinity College Dublin, Ireland; ^8^Institut de Neurociències & Department of Biochemistry and Molecular Biology, School of Medicine, Universitat Autònoma de Barcelona, Barcelona, Spain; and ^9^School of Chemical and Biomolecular Engineering, University of Sydney, Australia.

Corresponding Authors: James Curtin (james.curtin@TUDublin.ie) & Gillian Conway (gillian.conway@swansea.ac.uk)

**Running title:** Cold Atmospheric Plasma stimulates lysosome-associated cell death.

**Supplemental Materials 1**

**Table of data for figure 1a**

| **Time (hrs)** | **CAP 180sec (% viability)** | | | | | | | | | | | | | | |
| --- | --- | --- | --- | --- | --- | --- | --- | --- | --- | --- | --- | --- | --- | --- | --- |
| **0** | 98 | 108 | 106 | 103 | 104 | 98 | 105 | 100 | 102 | 102 | 94 | 90 | 100 | 98 | 92 |
| **1** | 65 | 125 | 126 | 84 | 97 | 101 | 88 | 85 | 79 | 65 | 125 | 126 | 84 |  |  |
| **4** | 33 | 75 | 70 | 55 | 36 | 29 | 82 | 105 | 64 | 33 | 40 | 33 | 75 | 70 | 55 |
| **8** | 28 | 29 | 30 | 30 | 29 | 23 | 30 | 27 | 33 | 27 | 29 | 28 | 29 | 30 | 30 |
| **21.5** | 20 | 15 | 18 | 20 | 25 | 21 | 14 | 16 | 16 | 18 | 19 | 20 | 15 | 18 | 20 |
| **33.5** | 23 | 28 | 33 | 31 | 29 | 26 | 24 | 20 | 25 | 25 | 23 | 23 | 28 | 33 | 31 |
| **45.5** | 28 | 32 | 35 | 36 | 30 | 21 | 36 | 28 | 25 | 22 | 28 | 32 | 35 | 36 |  |

**Table of data for figure 1d**

| **Time** | **% Membrane Permeabilization** | | | | | | | |
| --- | --- | --- | --- | --- | --- | --- | --- | --- |
| **Neg** | 11.3 | 7.43 | 8.35 | 7.7 | 14.11 | 6.64 | 12.88 | 8.52 |
| **0.5hr** | 48.78 | 53.43 | 41.9 | 50.19 |  |  |  |  |
| **2hr** | 57.99 | 68.79 | 58.58 | 72.46 |  |  |  |  |
| **4H** | 45.29 | 46.15 | 58.37 | 54.76 |  |  |  |  |
| **6hr** | 49.09 | 56.11 | 43.97 | 62.81 |  |  |  |  |
| **24hr** | 47.86 | 53.11 | 57.86 | 55.14 |  |  |  |  |
| **48hr** | 48.62 | 50.68 | 49.35 | 51.41 |  |  |  |  |
| **120hr** | 59.35 | 53.37 | 62.21 | 54.69 |  |  |  |  |

**Table and supplementary graph of data for figure 1e**

| **PI – Confocal quantification** | |
| --- | --- |
| **Untreated** | **APP** |
| 0. | 6438. |
| 0. | 22871. |
| 0. | 4716. |
| 0. | 29863. |
| 0. | 45658. |
| 0. | 24567. |
| 0. | 24303. |
| 0. | 22142. |
| 0. | 58906. |


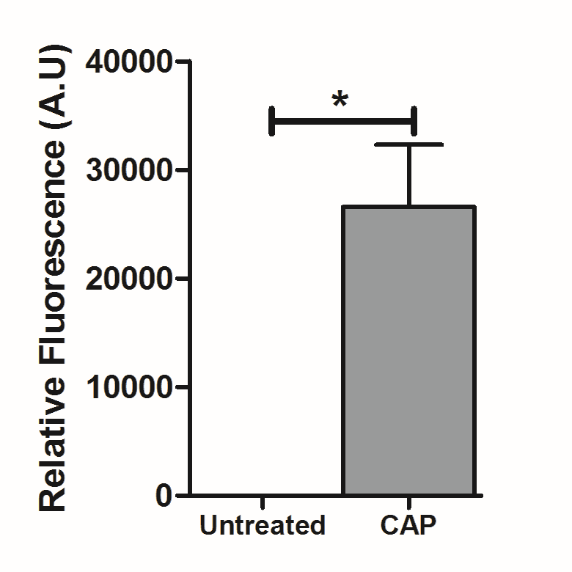


**Supplemental Material 2**

**Table of data for figure 2b**

| **JC-1 – Confocal quantification** | |
| --- | --- |
| **Untreated** | **CAP** |
| 47019.6500 | 8817.758 |
| 93285.6500 | 12109.000 |
| 60325.2100 | 53522.000 |
| 59240.0000 | 9644.000 |
| 33230.0000 | 7818.000 |
| 41930.0000 | 39623.000 |
| 37670.0000 | 8348.000 |
| 38750.0000 | 9593.000 |

| **Unt** | **CAP** | **NAC** | **zVAD** |
| --- | --- | --- | --- |
| 98. | 35. | 44. | 27. |
| 125. | 40. | 48. | 28. |
| 109. | 54. | 62. | 28. |
| 83. | 64. | 51. | 27. |
| 84. | 79. | 54. | 25. |
| 113. | 17. | 33. | 49. |
| 105. | 16. | 47. | 54. |
| 90. | 24. | 45. | 56. |
| 94. | 24. | 46. | 52. |
| 98. | 26. | 10. | 51. |
| 87. | 58. | 21. |  |
| 99. | 46. | 39. |  |
| 109. | 38. | 11. |  |
| 101. | 22. | 33. |  |
| 104. | 15. | 21. |  |
| 107. | 20. | 38. |  |
| 105. | 36. | 49. |  |
| 98. | 48. | 61. |  |
| 99. | 51. | 50. |  |

**Table of data for figure 2c**

**Supplemental Material 3**

**Supplementary Figure 3a**


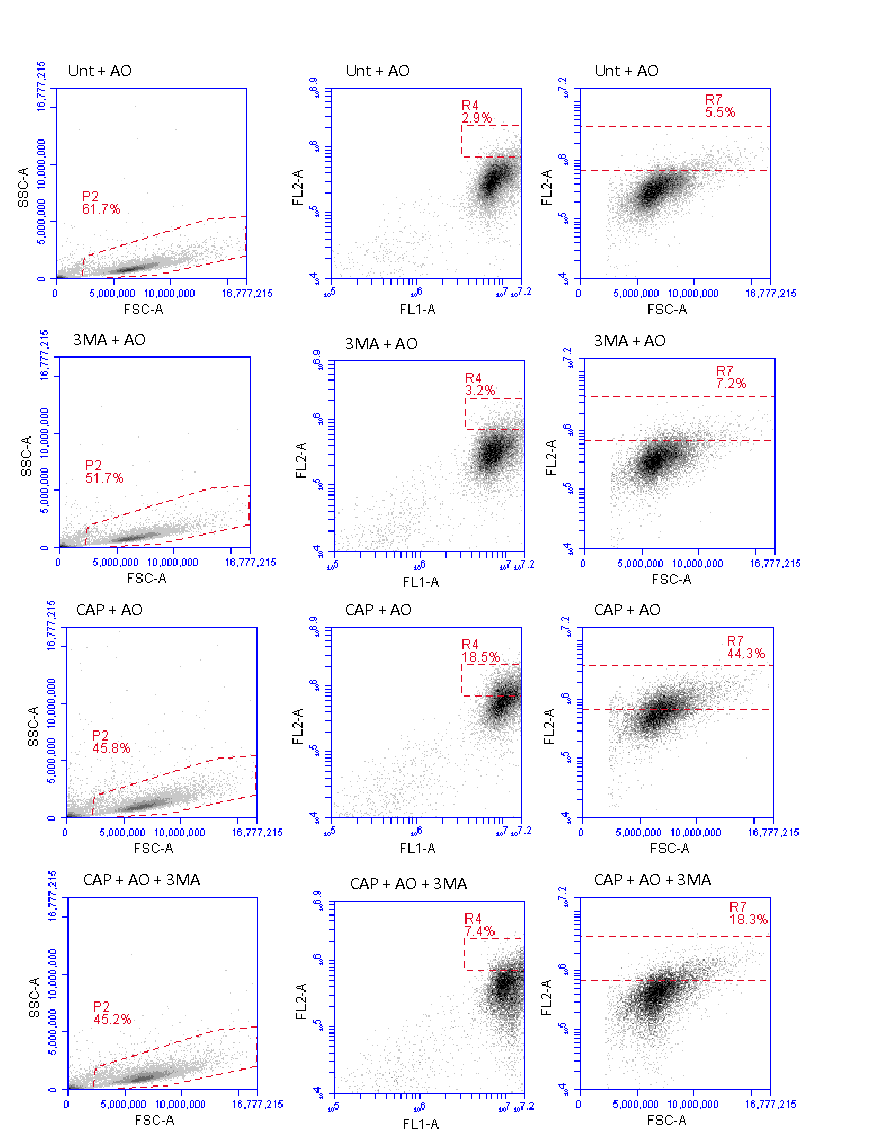


**Table of data for figure 3a**

| **Untreated** | **3-MA** | **CAP** | **CAP + 3-MA** |
| --- | --- | --- | --- |
| 0.4774128 | 0.5115706 | 1.445174 | 1.460987 |
| 1.217849 | 0.7253929 | 2.159349 | 1.569926 |
| 1.304743 | 1.393784 | 2.60051 | 1.830921 |
|  | 1.372213 |  | 1.701562 |

**Table of data for material 3b**

| **UNT** | **3-MA** | **CAP** | **3MA + CAP** |
| --- | --- | --- | --- |
| 465335. | 448723. | 113192. | 243547. |
| 455944. | 445475. | 171613. | 313936. |
| 441800. | 325751. | 208644. | 399889. |
| 428947. | 359100. | 210110. | 389974. |
| 485821. | 416189. | 151615. | 290717. |
| 537764. | 662690. | 267345. | 370539. |
| 554761. | 631872. | 326882. | 403319. |
| 543856. | 623490. | 301644. | 377025. |
| 533890. | 631479. | 340967. | 346085. |
| 541760. | 625812. | 360773. | 366136. |
| 481773. | 415516. | 171296. | 243587. |
| 301432. | 404150. | 214649. | 277885. |
| 367636. | 362040. | 164408. | 276826. |
| 330239. | 330606. | 157862. | 276100. |
| 479156. | 336924. | 221081. | 245505. |

**Supplemental Material 4**

**Supplementary figure 4a – Raw western Blot images**




**Supplementary figure 4b – Densitometry results from western blots.**
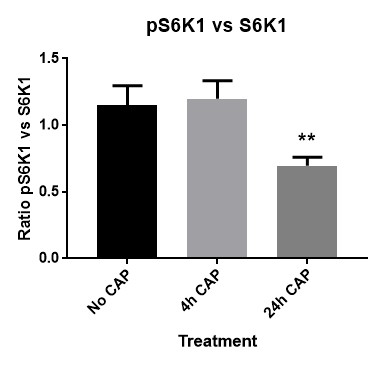


| **No CAP** | **4h CAP** | **24h CAP** |
| --- | --- | --- |
| 1.319017 | 1.350461 | 0.701196 |
| 1.057426 | 1.092086 | 0.757182 |
| 1.067393 | 1.150493 | 0.626939 |


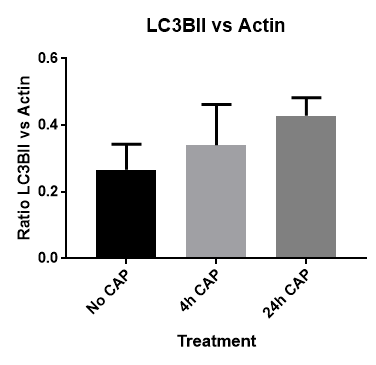


| **No CAP** | **4h CAP** | **24h CAP** |
| --- | --- | --- |
| 0.2579958 | 0.3196991 | 0.38402283 |
| 0.34681608 | 0.22951823 | 0.41174662 |
| 0.19379037 | 0.47144792 | 0.48914759 |


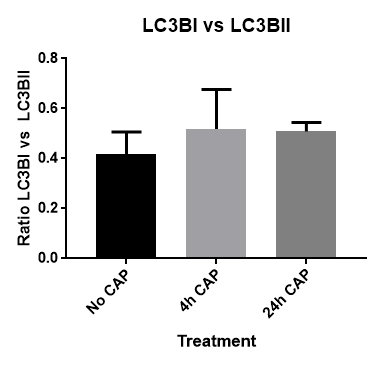


| **No CAP** | **4h CAP** | **24h CAP** |
| --- | --- | --- |
| 0.4681259 | 0.48242658 | 0.46744365 |
| 0.46907061 | 0.3823184 | 0.52865044 |
| 0.31388013 | 0.69118948 | 0.52935 |


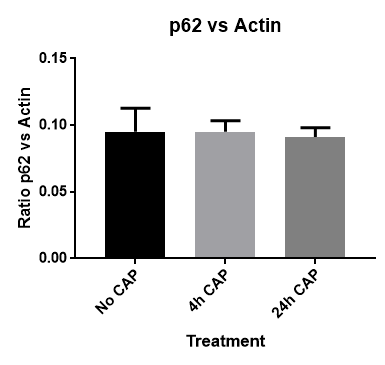


| **No CAP** | **4h CAP** | **24h CAP** |
| --- | --- | --- |
| 0.09375937 | 0.10441301 | 0.09810547 |
| 0.11329585 | 0.09175756 | 0.09129426 |
| 0.0781541 | 0.08941391 | 0.08400907 |

Supplementary Figure 4b Legend:

Band intensities were calculated using Photoshop and subtracted background. Non-saturation was confirmed using the histogram feature. Values are expressed as the integrals (area * mean density) of each band (normalized to actin).

Densitometry analysis confirms the levels of LC3BII and p62 do not change significantly following CAP treatment. We observed a small but significant decrease in pS6K1 after 24 hours but not after 4 hours, despite the presence of AVO's at this time point. Therefore, the reduction in pS6K1 at 24 hours is likely accounted by the loss of cell viability at this later time point to lead to mTORC1 inhibition. The delayed downregulation of Thr389 phosphorylation on S6K1 is likely due to the inactivation of mTORC1 which occurs during cell death.

**Supplemental Material 5**

**Table of data for figure 5a – Quantification of lysotracker Deep Red and Acridine orange staining**

| **IntDen of Green** | | **IntDen of Deep Red** | | **IntDen of AO** | |
| --- | --- | --- | --- | --- | --- |
| **Untreated** | **CAP** | **Untreated** | **CAP** | **Untreated** | **CAP** |
| 60776 | 49432 | 53054 | 68957 | 42124 | 110344 |
| 20949 | 35316 | 42069 | 89574 | 53300 | 59312 |
| 35181 | 22898 | 74079 | 90993 | 37271 | 45145 |
| 19745 | 26481 | 34059 | 92649 | 41267 | 49263 |
| 35884 | 47954 | 64455 | 105031 | 83687 | 40578 |
| 17014 | 22876 | 113445 | 29279 | 37621 | 70200 |
| 47770 | 21377 | 92708 | 29252 | 22375 | 38943 |
| 33108 | 25264 | 13819 | 56670 | 15439 | 39343 |
| 31551 | 72031 | 78105 | 119501 | 20790 | 61324 |
| 72824 | 20002 | 78768 | 54153 | 48477 | 90366 |
| 8346 | 32471 | 65613 | 66695 | 86673 | 50068 |
| 16916 | 27001 | 143435 | 152972 | 47268 | 112145 |
| 9535 | 24943 | 82036 | 175850 | 46623 | 77017 |
| 43872 | 38683 | 56611 | 65918 | 28274 | 62820 |
| 30881 | 57061 | 53877 | 170397 | 50449 | 91898 |
| 20015 | 27716 | 45964 | 143379 | 34575 | 77137 |
| 18799 | 55997 | 51604 | 40998 | 46628 | 40108 |
| 18385 | 17542 | 32886 | 99572 | 55599 | 100278 |
| 19113 | 29450 | 34575 | 158774 | 42260 | 48492 |
| 17378 | 19404 | 55934 | 124630 | 40078 | 81911 |
| 34289 | 26130 | 29564 | 34824 | 44941 | 79228 |
| 33159 | 9539 | 29737 | 90747 | 45063 | 71910 |
| 22108 | 54974 | 25610 | 108508 | 39387 | 51817 |
| 34354 | 36920 | 38336 | 55367 | 37910 | 50926 |
| 137971 | 23065 | 60252 | 66859 | 40993 | 52202 |
| 19445 | 11973 | 33342 | 234083 | 28517 | 72828 |
| 24250 | 44464 | 54320 | 109460 | 86964 | 84712 |
| 54976 | 23764 | 84042 | 73315 | 52790 | 63514 |
| 18242 | 62208 | 48641 | 66851 | 47377 | 94162 |
| 15036 | 25811 | 120796 | 34407 | 67104 | 199606 |
| 31510 | 32396 | 76078 | 72587 | 23253 | 69853 |
| 30857 | 14229 | 107685 | 22422 | 26573 | 81642 |
| 21840 | 86810 | 110760 | 29401 | 14991 | 94606 |
| 18848 | 8763 | 119957 | 33715 | 14893 | 87768 |
| 52308 | 43664 | 41939 | 15562 | 16949 | 88027 |
| 19845 | 38703 | 127778 | 27811 | 5918 | 79333 |
| 19210 | 54728 | 62744 | 107938 | 6961 | 147225 |
| 13726 | 27130 | 55334 | 29401 | 6085 | 41638 |
| 13030 | 16903 | 23385 | 123622 | 25628 | 74409 |
| 12412 | 14745 | 72125 | 48163 | 6466 | 68562 |
| 37509 | 46133 | 30717 | 39012 | 16374 | 40176 |
| 25057 | 23916 | 40988 | 70869 | 11911 | 36140 |
| 18177 | 18506 | 46013 | 68320 | 61149 | 124616 |
| 10613 | 9320 | 26298 | 53931 | 40407 | 72313 |
| 63417 | 14939 | 9519 | 137881 | 56504 | 110813 |
| 58644 | 21067 | 34372 | 118110 | 17963 | 71248 |
| 63947 | 62873 | 34453 | 89251 | 32226 | 175716 |
| 29522 | 48029 | 41959 | 302409 | 25575 | 77221 |
| 65171 | 18928 | 57701 | 90330 | 54936 | 41179 |
| 46827 | 28000 | 58031 | 197917 | 58872 | 87922 |
| 26179 | 18243 | 24595 | 20151 | 23633 | 26726 |
| 22712 | 28574 | 25097 | 309776 | 47533 | 88805 |
| 26588 | 71695 | 89000 | 116160 | 24862 | 108769 |
| 53064 | 40976 | 28761 | 41533 | 13570 | 165815 |
| 15298 | 42889 | 57348 | 133129 | 5891 | 61772 |
| 23110 | 34278 | 122010 | 53467 | 10771 | 54676 |
| 45287 | 38963 | 36793 | 89070 | 15812 | 36793 |
| 19527 | 45536 | 21282 | 140004 | 11938 | 21282 |
| 9190 | 23912 | 83995 | 43655 | 15857 | 83995 |
| 61962 | 10402 | 68541 | 110696 | 5411 | 68541 |
| 23843 | 10819 | 66625 | 34793 | 31139 | 66625 |
| 51126 | 7259 | 31401 | 54349 | 3577 | 31401 |
| 80833 | 36776 | 35281 | 33894 | 22844 | 35281 |
|  |  | 90267 | 39083 | 27412 | 90267 |
|  |  | 78259 | 102074 | 52376 | 78259 |
|  |  | 123186 | 66511 | 92060 | 123186 |
|  |  | 116189 | 156145 | 31989 | 116189 |
|  |  | 29066 | 133499 | 11738 | 29066 |
|  |  | 62434 | 202604 | 40177 | 62434 |
|  |  | 34182 | 59965 | 61116 | 34182 |
|  |  | 74690 | 18991 | 22741 | 74690 |
|  |  | 38866 | 136934 | 39809 | 38866 |
|  |  | 19420 | 114607 | 12193 | 19420 |
|  |  | 63121 | 59478 | 54804 | 63121 |
|  |  | 64328 | 199464 | 122053 | 64328 |
|  |  | 17919 | 200675 | 60171 | 17919 |
|  |  | 85423 | 200993 | 34925 | 85423 |
|  |  | 38887 | 49412 | 30025 | 38887 |
|  |  | 47278 | 72776 | 38627 | 47278 |
|  |  | 25915 | 249786 | 48605 | 25915 |
|  |  | 16634 | 173126 | 108116 | 16634 |
|  |  | 86326 | 104034 | 30174 | 86326 |
|  |  | 63361 | 52259 | 10587 | 63361 |
|  |  | 67238 | 203079 | 19468 | 67238 |
|  |  | 65834 | 159826 | 35100 | 65834 |
|  |  | 59583 | 50176 | 55649 | 59583 |
|  |  | 43109 | 157005 | 18736 | 43109 |
|  |  | 67682 | 27850 | 65216 | 67682 |
|  |  | 17865 | 154808 | 72229 | 17865 |
|  |  | 41529 | 70604 |  | 41529 |
|  |  | 83825 | 65216 |  | 83825 |
|  |  | 24587 | 72229 |  | 24587 |
|  |  | 42703 |  |  | 42703 |
|  |  | 51514 |  |  | 51514 |
|  |  | 13705 |  |  | 13705 |
|  |  | 122181 |  |  | 122181 |
|  |  | 17453 |  |  | 17453 |
|  |  | 111481 |  |  | 111481 |
|  |  | 26950 |  |  | 26950 |
|  |  | 16820 |  |  | 16820 |
|  |  | 44200 |  |  | 44200 |
|  |  | 69937 |  |  | 69937 |
|  |  | 12686 |  |  | 12686 |
|  |  | 24002 |  |  | 24002 |
|  |  | 31863 |  |  | 31863 |
|  |  | 42893 |  |  | 42893 |
|  |  | 18182 |  |  | 18182 |
|  |  | 32601 |  |  | 32601 |
|  |  | 64669 |  |  | 64669 |
|  |  | 33625 |  |  | 33625 |
|  |  | 40387 |  |  | 40387 |

**Supplemental Figure 6 -**



**AVO formation in A549 and HeLa cells following CAP treatment.** *In-situ* verification of AVO’s was measured in 1µg/ml Acridine Orange (AO) stained A549 cells (60 seconds) and HeLa cells (5 seconds) 24hr after CAP exposure (75kV). Integrated density of AO staining was determined using confocal microscopy for at least 95 cells in each sample. Significant differences between untreated and CAP treated cells were determined using an unpaired two-tailed t-test (**p<0.01, ****p<0.0001).

**Supplemental figure 7 – Characterisation DT120 CAP device.**

**To identify the presence of ROS formation following CAP treatment**. U373MG cells were exposed to CAP for 180 seconds at 75kV both in the presence and absence of cell culture media containing increasing concentrations of pyruvate (a well-known reactive oxygen species scavenger). Statistical analysis was carried out using One-way ANOVA with Bonferroni post-test (*P<0.001). As seen in supplemental figure 7, there is a correlation between increasing concentrations of pyruvate with increases in cell viability, thus providing evidence for the presence of ROS formation and ROS-dependent cytotoxicity following CAP treatment*.*

**Supplemental figure 8**

**Supplementary figure 8a Positive Control for Caspase-dependent Apoptosis (related to figure 2c).**


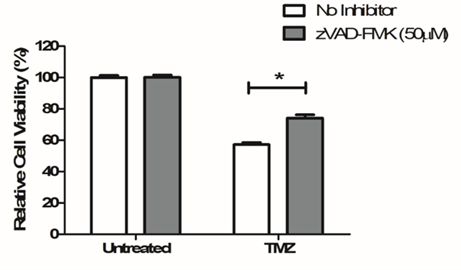


**Supplementary figure 8b Dose response of zVAD-FMK measuring cytotoxicity (related to figure 2c).**


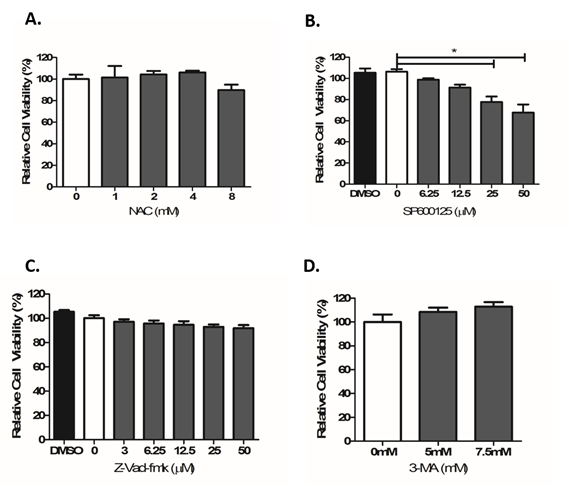

Supplement: Supplementary file 1 — Supplemental Material [file 41598_2019_49013_MOESM1_ESM.docx]
